# B-cell immune checkpoint TIM-1: a potential target for tumour immunotherapy

**DOI:** 10.1038/s41392-023-01643-w

**Published:** 2023-10-20

**Authors:** Xinyu Tian, Xiangyun Zheng, Dong Tian

**Affiliations:** 1 Department of Thoracic Surgery, West China Hospital, Sichuan University, 37 Guoxue Alley, Chengdu, 610041 China; 2 Lung Transplant Research Laboratory, Institute of Thoracic Oncology, West China Hospital, Sichuan University, 37 Guoxue Alley, Chengdu, 610041 China

**Keywords:** Drug development, Tumour immunology

Recently, Lloyd Bod et al. published a study in *Nature* that identified the B-cell immune checkpoint T cell immunoglobulin and mucin domain 1 (TIM-1) and mechanistically elucidated that targeting TIM-1 enhances the responses to type I interferon (IFN-I), promotes B cells antigen presentation and activation, subsequently enhances anti-tumour responses of CD4^+^ and CD8^+^ T cells and inhibits tumour growth. This study provides important implications for anti-tumour therapy.^1^

Tumour immunotherapy, such as treatment with immune checkpoint blockade (ICB), has revolutionised the approach to dealing with tumours and has significantly improved long-term survival rates.^2^ Currently, immune checkpoint inhibitors (ICIs) have made significant advancements in anti-tumour clinical applications, representing a breakthrough in the field of tumour therapy.^3^ Some of the well-established ICIs include cytotoxic T lymphocyte-associated protein 4 (CTLA-4), programmed death 1 (PD-1) and programmed death-ligand 1 (PD-L1). Additionally, there are several ICIs currently undergoing preclinical or clinical studies, such as cluster of differentiation 47 (CD47), T cell immunoglobulin domain and mucin domain 3 (TIM-3), and lymphocyte-activation gene 3 (LAG-3).^4^ However, not all patients respond to immunotherapy, which highlights the need to identify specific mechanisms and biomarkers that can enhance its effectiveness and application.

While boosting T cells and NK cells function has primarily been the focus of tumour immunotherapy, the function of B cells, a crucial component of the immune system, in anti-tumour immunity remains a subject of debate.^1^ TIM-1, expressed on a subset of peripheral B cells, binds to phosphatidylserine exposed on apoptotic cells to promote tissue tolerance.^1^ However, its potential role in cancer has not been thoroughly investigated.

To investigate how B cell subsets regulate anti-tumour immune responses, B cells were globally depleted by anti-CD20 monoclonal antibodies, leading to an increase in B16F10 melanoma tumour growth. Moreover, it was observed that the expression profiles of tumour-infiltrating B cells were specific through RNA-sequencing (RNA-seq). These findings suggest that the presence of total B cells possesses tumour-suppressive properties and showed a unique phenotype upon infiltration in melanoma tumour.

Furthermore, to investigate total B cell heterogeneity, CD45^+^ cells in tumour microenvironment (TME), draining lymph nodes (dLN), and non-draining lymph nodes (ndLN) were analysed during growth of B16F10 melanoma tumour. Then, the population of B cell that expanded in tumour, dLN and ndLN or over time were searched based on transcriptional states or B cell receptor (BCR) clones. The results revealed that the frequency of a specific cluster of B cells in dLN cells increased with tumour growth, which suggests that B16F10 melanoma tumour growth induces a unique subset of B cells. Furthermore, by combining COMET, flow cytometry analysis and bulk RNA-seq, the authors found an overexpression of the genes associated with the activation and proliferation of B cells in TIM-1^+^ B cell subset from dLN. Additionally, B cells bearing TIM-1 from mice with B16F10 melanoma tumour exhibited higher expression levels of synergistic inhibitory and immunoregulatory factors that are commonly found on T cells. Then, to investigate how these factors impact the anti-tumour immunity, selected removal of TIM-1 was conducted. The results demonstrate that the loss of *Havcr1*, which encodes TIM-1, significantly inhibits tumour growth in various tumour models, highlighting the importance of TIM-1 expressing B cells in suppressing tumour growth.

Next, to validate how targeting TIM-1 regulates tumour growth, a high-affinity anti-TIM-1 antibody (3B3) was administered to B16F10 mice. It was observed that 3B3 did not exhibit therapeutic effect in μMT mice or *hCD20.TamCre* *×* *Havcr1*^*fl/fl*^ (*Havcr1*^*BKO*^) mice, which indicates that the presence of B cells is a prerequisite for the therapeutic effect of anti-TIM-1 antibody. Moreover, treating the spontaneous melanoma model, *Tyr-cre*^*ERT2*^*Bra*^*fCA/WT*^*Pten*^*lox/lox*^ mice, with the anti-TIM-1 antibody resulted in a significant reduction of tumour growth. Further, combining PD-1 blockade with anti-TIM-1 antibody resulted in a more effective inhibition of tumour growth. Notably, treatment with the anti-TIM-1 antibody alone or with PD-1 blockade increased the fraction of activated CD4^+^ and CD8^+^ T cells. These findings indicated that targeting TIM-1 therapeutically reduces tumour growth and requires B cells expressing TIM-1.

Besides, to investigate the influence of TIM-1 expressing B cells on regulating tumour growth, the flow cytometry analysis to CD45^+^ cells was performed in dLN, ndLN and TME after the inoculation of B16F10 cells in *Havcr1*^*BKO*^ mice. Additionally, the percentage of activated CD4^+^and CD8^+^ T cells within tumour-infiltrating leucocytes (TILs) from *Havcr1*^*BKO*^ mice were higher than those of control mice. Moreover, CD45^+^ cells from dLN, ndLN and TME were profiled using single-cell RNA- and TCR-seq (scRNA/TCR-seq). It was confirmed that the clonal expansion and tumour infiltration of CD8^+^ T cells were enhanced in *Havcr1*^*BKO*^ tumours. Interestingly, clonally amplified CD8^+^ T cells from *Havcr1*^*BKO*^ tumours have effector/cytotoxic phenotype-associated genes expression. Meanwhile, staining with H-2K^b^-OVA_257–264_ dextramer and Ki-67 expression of TILs from mice with B16-OVA demonstrated a higher proliferation of OVA-specific CD8^+^ T cells in *Havcr1*^*BKO*^ tumour than that from control group.

Additionally, to investigate the regulatory function of B cell loss in the anti-tumour responses mediated by T cells, *Havcr1* deletion was performed in B cells. The scRNA-seq analysis of *Havcr1*^*BKO*^ B cells from tumours and dLNs indicated a remarkable improvement for BCR sensing, activation, and immune co-stimulation gene signatures of B cells, along with increased expression of characteristics associated with IFN response. The results suggest that the deletion of *Havcr1* had minimal impact on humoral immunity. Furthermore, to investigate how the genetic deletion of *Havcr1* affected antigen presentation of B cells to CD4^+^ T cells, the proliferating frequency of T cells was determined. As the results show, a higher level of T cell proliferation was found to be induced by *Havcr1*^*BKO*^ B cells in MHC II presentation-dependent manner.

Moreover, the impact of *Havcr1*^*BKO*^ B cells on T cells was assessed by analysing the expression levels of IFNγ, ICOS, and FOXP3. The analysis revealed that a larger percentage of IFNγ^+^ cells and a substantial increased expression of ICOS were induced by *Havcr1*^*BKO*^ B cells promote the expression of IFNγ and ICOS in CD4^+^ T cells, while inhibit the expression of FOXP3. In addition, naive CD4^+^ T cells from CD45.1 OT-II donors were adoptively transferred into congenic CD45.2 *Havcr1*^*BKO*^, in which tumour-derived CD45.1^+^CD4^+^ T cells showed increased IFNγ expression and reduced FOXP3 expression. These findings indicate that TIM-1 could suppress antigen presentation by B cells. Notably, ex vivo *Havcr1*^*BKO*^ B cells were found to exhibit an IFN-I gene signature, which is associated with increased responsiveness to IFN-β and enhanced IFNα/β receptor (IFNAR) expression. Additionally, blocking IFNAR1 reversed the tumour growth control in *Havcr1*^*BKO*^ mice. These two findings indicate that the expression of TIM-1 is regulated by both IFN-I and IFN-II, and loss of TIM-1 enhances the sensing of IFN-I and IFN-II by B cells.

**Fig. 1:**
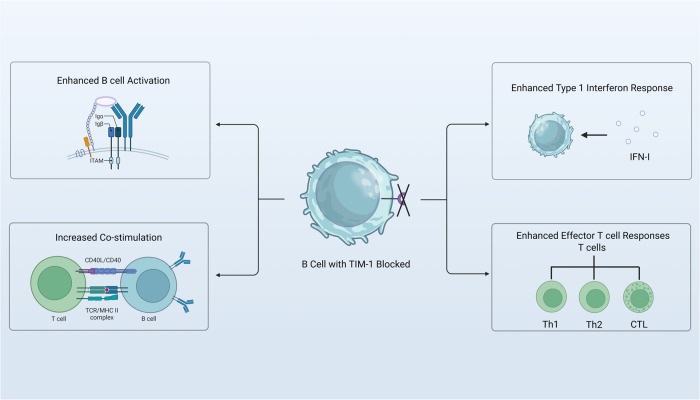
TIM-1 blockade in B cells enhances the sensing of IFN by B cells. This leads to an augmented IFN-I response, increased B cell activation, enhanced antigen presentation, and improved co-stimulation. Consequently, there is an increase in inflammatory cytokine production and responsiveness, resulting in enhanced effector T cell responses. Created with BioRender.com

In summary, this study suggests that TIM-1 functions as an immune checkpoint to B cell activation. Furthermore, deficiency of TIM-1 leads to an upregulation of B cells IFN-I response, resulting in enhanced activation, antigen presentation of B cells and immune co-stimulation (Fig. 1). Activation of the adaptive immune system by blocking TIM-1 can potentially improve the effectiveness of cancer immunotherapy and provides novel strategies for immune check point blockade.
